# Single, but not dual, attention facilitates statistical learning of two concurrent auditory sequences

**DOI:** 10.1038/s41598-017-10476-x

**Published:** 2017-08-31

**Authors:** Tatsuya Daikoku, Masato Yumoto

**Affiliations:** 1 Department of Clinical Laboratory, Graduate School of Medicine, The University of Tokyo, Tokyo, Japan; 2 Department of Neuropsychology, Max Planck Institute for Human Cognitive and Brain Sciences, Leipzig, Germany

## Abstract

When we are exposed to a novel stimulus sequence, we can learn the sequence by extracting a statistical structure that is potentially embedded in the sequence. This mechanism is called statistical learning, and is considered a fundamental and domain-general process that is innate in humans. In the real-world environment, humans are inevitably exposed to auditory sequences that often overlap with one another, such as speech sound streams from multiple speakers or entangled melody lines generated by multiple instruments. The present study investigated how single and dual attention modulates brain activity, reflecting statistical learning when two auditory sequences were presented simultaneously. The results demonstrated that the effect of statistical learning had more pronounced neural activity when listeners paid attention to only one sequence and ignored the other, rather than paying attention to both sequences. Biased attention may thus be an essential strategy when learners are exposed to multiple information streams.

## Introduction

The brain is a learning system that adapts to multiple stimuli in a living environment. Learners can acquire a great deal of information attentionally and nonattentionally^1, 2^. Statistical learning, which is a learning system of transitional probabilities in sequential information such as music and language, has been hypothesised to be a fundamental, domain-general, and automatic process regardless of attention^3–9^. Several studies demonstrated that the statistical learning of tone sequences could be reflected in neural responses elicited in the auditory cortex^10–17^; auditory responses to tones that appeared with a higher transitional probability were reduced compared to those with a lower transitional probability, while listeners acquired statistical knowledge from the exposed tone sequence. However, a previous study suggests that, based on the implicitness of statistical learning in humans, we cannot delineate learned statistical knowledge, even when we tried to “intentionally” learn statistical knowledge^13^. Although humans are unaware of the learned statistical structure of auditory sequences, they can, instead, recognize sequences with at least nine tones transitioned with high probability as familiar tone sequences^13, 14, 16, 17^. Thus, statistical knowledge itself is implicit and does not reach the level of explicit awareness, whereas it can be alternatively expressed via an abstract medium such as a musical melody.

Using a Markov chain^11, 13, 14, 16, 17^ or a word segmentation task^10, 12, 15, 17^, previous studies demonstrated that auditory statistical learning could be reflected in auditory event-related responses, such as P1/P1 m^12, 16^, N1/N1 m or mismatch negativity/mismatch field^10–15, 17^, and P2/P2 m^11^, peaking in a latency range from 50 to 200 ms after stimulus onset. Some studies suggest that the learning effect relationship with P1 m involves music expertise and specialised training experience^12, 16^. When pure tone sequences were presented, learning effects on P1 m, but not N1 m, were larger in musicians compared with non-musicians^12^. In our previous study, the statistical learning of atonal chord sequences of augmented triads was reflected in P1 m^16^. However, when auditory sequences of complex tones were presented, statistical learning was reflected in N1 m^13, 14, 17^. Thus, the type of the stimulus, the structural character of the stream, attention to the stimuli, and the instructions given to the listeners may modulate neural responses for statistical learning in the auditory cortex. However, the relationships between event-related responses elicited in the auditory cortex and statistical learning effect is still controversial.

Despite nonattentional sensitivity to statistics in humans, we often attentionally access linguistic and musical knowledge that potentially involves statistical information. A previous neurophysiological study on auditory statistical learning demonstrated that attentional statistical learning could be more effective than nonattentional statistical learning^13^. This suggests that attention to the auditory stimuli modulates the neural basis underlying statistical learning in the auditory cortex. According to the predictive coding theory^18^, the brain constantly generates probabilistic predictions based on learned knowledge. When a predicted tone is presented, there is a suppression of activities in the primary auditory cortex. In the previous studies on auditory statistical learning, auditory responses to predictable tones were reduced compared to unpredictable tones. Thus, there might be relationships between attention and prediction in auditory mechanisms underlying statistical learning. It is necessary to verify how neural responses in the auditory cortex are modulated by attention, in relation to prediction during auditory statistical learning.

Given the superiority of the attentional strategy over the nonattentional strategy for statistical learning, it is not known why we possess nonattentional learning ability. It might be difficult to pay attention to every detail of multiple information streams that can occur concurrently as a result of cognitive capacity limitations in humans^19^. Our living environment, however, is rich in overlapping sound streams, such as human voices, musical instruments, water, and wind. In general, learners can acquire a great deal of information through both attentional and nonattentional processes^1, 2^. A previous study behaviourally demonstrated that attentional and nonattentional learning operated independently and in parallel when learners were presented with two simultaneous streams of stimuli^20^. Thus, nonattentional learning may be necessary when learners are simultaneously exposed to multiple pieces of auditory information. Few studies have neurophysiologically investigated nonattentional and attentional statistical learning when humans are simultaneously exposed to multiple auditory streams of statistically structured sequences. In the present study, we used magnetoencephalography (MEG) to investigate how attentional and nonattentional learning were reflected in neurological responses in the auditory cortex when participants were presented with two simultaneous streams of tones.

## Results

### Experimental procedure

**Figure 1:**
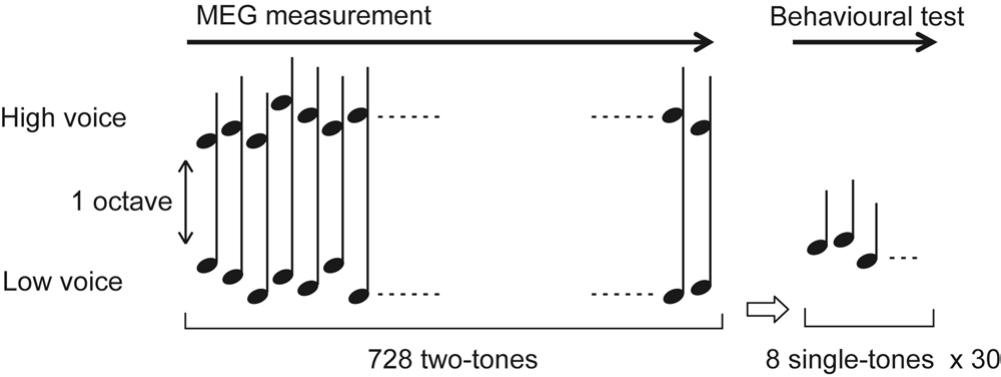
Experimental procedure. The dyad sequences with 728 two-tones can also be interpreted as two simultaneous sequences that consisted of low- and high-voice sequences. After measuring MEG, participants completed an interview in which they were presented with 30 series of eight single tones.

**Figure 2:**
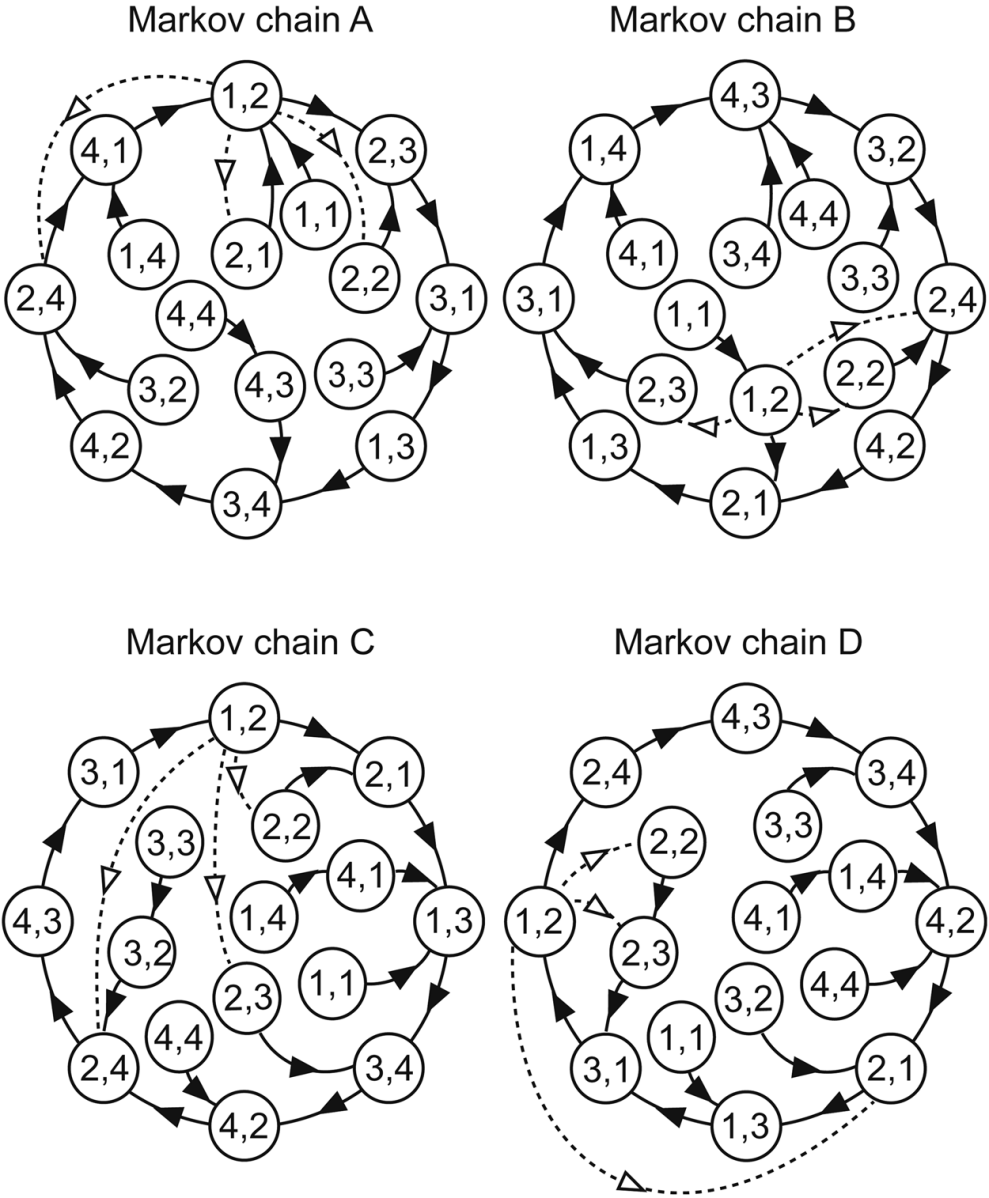
State transition diagrams of the Markov chains used in the present study. The paired digits in the circles represent two successive tones in the stimulus sequence. A forthcoming tone was statistically defined by the last two successive tones. The distinct two of four Markov chains were used in each of the low and high voices, and the use of Markov chains was counterbalanced across participants. The solid arrows represent transitions from each state with a high probability (80%). The remaining possible transitions from each state to the other three states occurred with a low probability (6.67% each); the low probability transitions only from the state (1, 2) are shown by dashed arrows to avoid illegibility.

The experiment consisted of two sessions. In each session, MEG was carried out on the participants while they were listening to a dyad (two-note chord) sequence, and learning achievement was evaluated using behavioural tests (Fig. 1). The dyad sequences can be separated into high- and low-voiced concurrent tone sequences, within which the intervals were separated by more than an octave and presented every 0.5 s. The tone transition in high- and low-voice sequences was independently ruled by distinct second-order Markov chains, such that a forthcoming tone was statistically defined by the latest two successive tones in each voice (Fig. 2). Hereafter, the tones that appeared with higher and lower transitional probabilities are termed frequent and rare tones, respectively.

In the single attention session, participants were instructed to listen to either the high- or low-voice sequence (attended sequence) and ignore the other sequence (ignored sequence). After MEG measurement, participants completed an interview in which they were presented with 30 series of 8 tones. Participants then reported whether each 8-tone series sounded familiar. The 30 series of 8 tones were categorised into three types, and the presentation order was randomised. In the 10 series, tones were ordered based on the same constraint as the ignored sequence (tone series I). In an additional 10 series, tones were ordered based on the same constraint as the attended sequence (tone series A). In the remaining 10 series, tones were pseudo-randomly ordered (random tone series). The target of analysis was the MEG responses to the dyads, which were categorised into four groups: 2 (frequent, rare) × 2 (attended and ignored sequences).

In the dual attention session, participants were instructed to listen to both the high- and low-voice sequences. After MEG measurement, participants completed an interview in which they were presented with 30 series of 8 tones. Participants then reported whether each 8-tone series sounded familiar. The 30 series of 8 tones were categorised into three types, and the presentation order was randomised. In the 10 series, tones were ordered based on the same constraint as the high-voice sequence (high-voice series). In an additional 10 series, tones were ordered based on the same constraint as the low-voice sequences (low-voice series). In the remaining 10 series, tones were pseudo-randomly ordered (random tone series). The target of analysis was the MEG responses to the dyads, which were categorised into four groups: 2 (frequent, rare) × 2 (high- and low-voiced).

### Behavioural results

**Figure 3:**
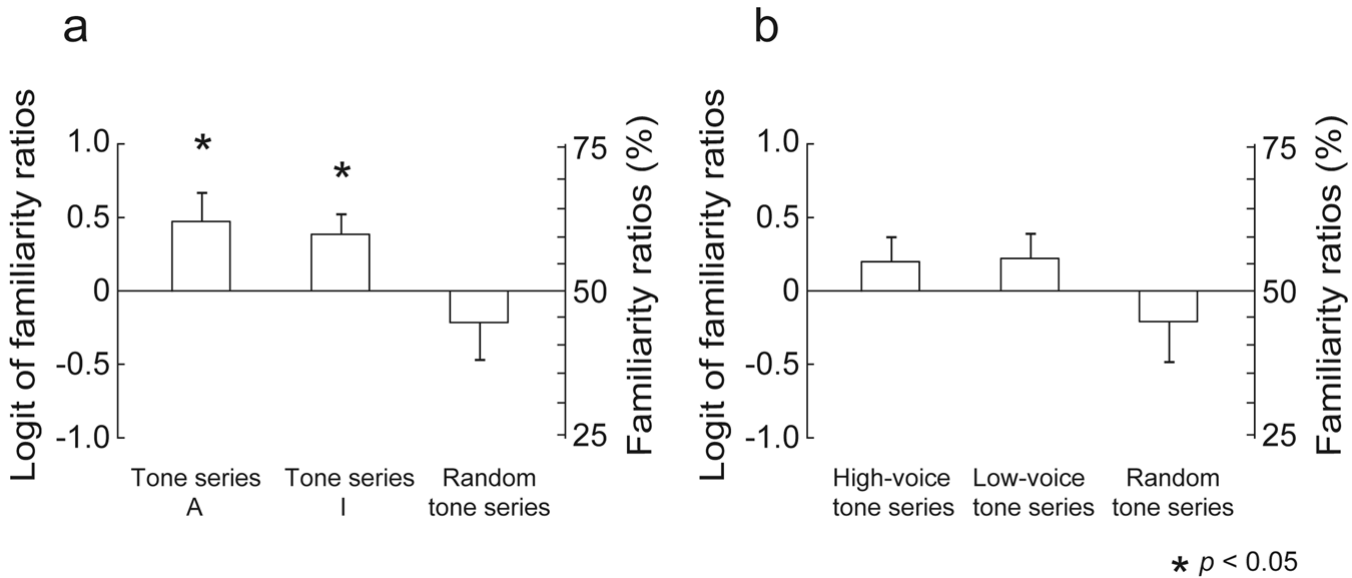
Results of answering that the tone series sounded familiar in the (**a**) single and (**b**) dual attention sessions. (**a**) In tone series A, tones were ordered based on the same constraint as the attended sequence. In tone series I, tones were ordered based on the same constraint as the ignored sequence. In the random tone series, tones were pseudo-randomly ordered. (**b**) In the high-voice series, tones were ordered based on the same constraint as for the high-voice sequence. In the low-voice tone series, tones were ordered based on the same constraint as in the low-voice sequences. In the random tone series, tones were pseudo-randomly ordered. The bars indicate the standard error of the mean. Only the tone series A and I in the single attention session significantly sounded familiar to the participants. In the single attention session (**a**), the familiarity ratios were significantly above the level of chance (50%) in both tone series A and I. In the dual attention session (**b**), the familiarity ratios were not significantly different from the level of chance in any type of tone series.

In the single attention session, the results of two-tailed *t*-tests indicated that the familiarity ratios were significantly above chance level in both tone series A and I (tone series A: t[14] = 2.74, *p* = 0.016; tone series I: t[14] = 2.72, *p* = 0.017) (Fig. 3). The analysis of variance (ANOVA) for the single attention session detected a significant difference (*F*[2, 28] = 4.79, *p* = 0.016). The Bonferroni-corrected post-hoc test revealed that there was no significant difference. In the dual attention session, the results of two-tailed *t*-tests indicated that the familiarity ratios were not significantly different from the level of chance in any type of tone series. No other significant results were detected in the behavioural tests.

### Magnetoencephalographic results

**Figure 4:**
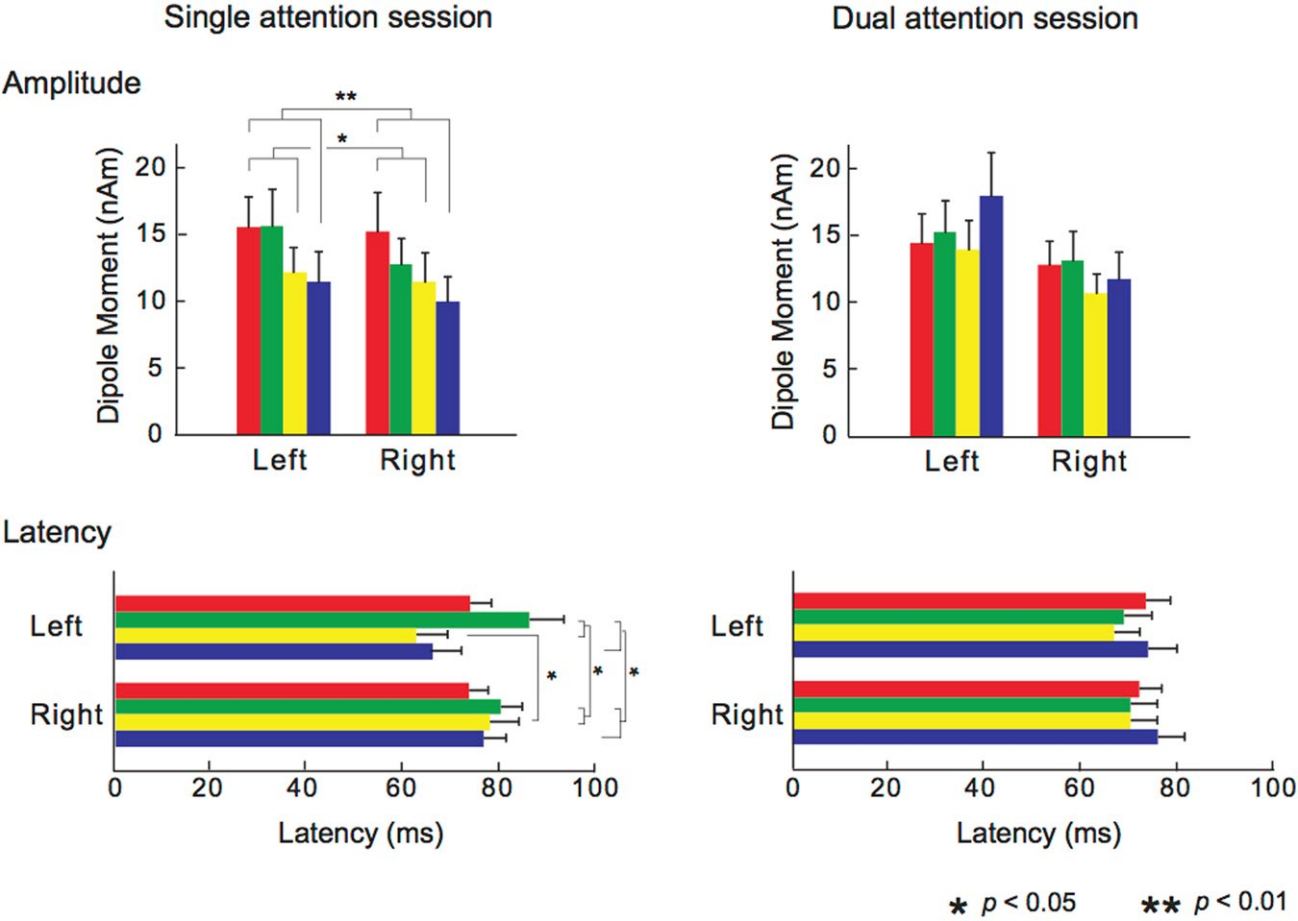
Grand-averaged peak amplitudes and latencies of source-strength waveforms for the P1 m responses (n = 12). The single and dual attention sessions are located on the left and right sides, respectively. Single attention session: the red bars represent the responses to dyads that consisted of two rare tones in both the ignored and attended sequences; green bars represent the responses to dyads that consisted of a frequent tone in the ignored sequence and a rare tone in the attended sequence; yellow bars represent the responses to dyads that consisted of a rare tone in the ignored sequence and a frequent tone in the attended sequence; and blue bars represent the responses to dyads that consisted of two frequent tones in both the ignored and attended sequences. Dual attention session: the red bars represent the responses to dyads that consisted of two rare tones in both the high- and low-voice sequences; green bars represent the responses to dyads that consisted of a rare tone in the high-voice sequence and a frequent tone in the low-voice sequence; yellow bars represent the responses to dyads that consisted of a rare tone in the low-voice sequence and a frequent tone in the high-voice sequence; and blue bars represent the responses to dyads that consisted of two frequent tones in both the high- and low-voice sequences. In the single attention session, the peak amplitudes for the dyads that consisted of two rare tones in both the ignored and attended sequences were significantly increased compared with those for dyads that consisted of a rare tone in the ignored sequence and a frequent tone in the attended sequence, and also those for the dyads that consisted of two frequent tones in both the ignored and attended sequences. However, in the dual attention session, no other significant differences were detected.

The averaged amplitudes and latencies of P1 m are shown in Fig. 4. Because there were insufficient samples of N1 m and P2 m for statistical analysis based on the criteria of equivalent current dipole (ECD) estimation with a goodness-of-fit above 80%, N1 m and P2 m components were excluded from further analysis. In the single attention session, the main stimulus effect on the P1 m peak amplitudes and latencies were significant (amplitudes *F*[3, 33] = 5.62, *p* = 0.0030; latencies *F*[3, 33] = 4.52, *p* = 0.0092). The P1 m peak amplitudes for the dyads that consisted of two rare tones in both the ignored and attended sequences were significantly increased compared with those for dyads that consisted of a rare tone in the ignored sequence and a frequent tone in the attended sequence (*p* = 0.010), and also those for the dyads that consisted of two frequent tones in both the ignored and attended sequences (*p* = 0.0037; Fig. 4). The P1 m peak latencies for the dyads that consisted of a frequent tone in the ignored sequence and a rare tone in the attended sequence were significantly longer compared with those for the dyads that consisted of a rare tone in the ignored sequence and a frequent tone in the attended sequence (*p* = 0.042), and also those for the dyads that consisted of two frequent tones in both the ignored and attended sequences (*p* = 0.042; Fig. 4). The hemisphere-tone interactions of the P1 m peak latencies were significant (*F*[3, 33] = 3.83, *p* = 0.019). In the dyads that consisted of a rare tone in the ignored sequence and a frequent tone in the attended sequence, the P1 m peak latencies were significantly shorter in the left than the right hemispheres (*p* = 0.022). In the left hemisphere, the P1 m peak latencies for the dyads that consisted of a frequent tone in the ignored sequence and a rare tone in the attended sequence were significantly longer compared with those for dyads that consisted of a rare tone in the ignored sequence and a frequent tone in the attended sequence (*p* = 0.014), and also those for the dyads that consisted of two frequent tones in both the ignored and attended sequences (*p* = 0.023). In the dual attention session, no other significant differences were detected.

## Discussion

By learning statistics of transitional probabilities embedded in tone sequences, learners can predict a tone that will follow certain preceding tones in the sequence. With this prediction for upcoming tones, tones with higher transitional probability (i.e., more predictable tones) lead to a decrease in amplitude and shortening of latencies in neural responses. In contrast, tones with lower transitional probability (i.e., less predictable tones) lead to an increased neural response amplitude^10–17^. In the present study, participants were presented with two simultaneous tone sequences that had tones with higher and lower transitional probabilities (i.e., frequent and rare tones, respectively). Based on the combinations of frequent and rare tones in the two simultaneous tone sequences, there were four types of dyads: a dyad that consisted of two frequent tones in both sequences, a dyad that consisted of two rare tones in both sequences, a dyad that consisted of a frequent tone in a sequence and a rare tone in the other sequence, and vice versa. If participants could acquire statistical knowledge in the two tone sequences, the dyad that consisted of two frequent tones should have the lowest amplitudes, and those consisting of two rare tones should have the highest amplitudes. In contrast, the increase in responses to the dyad that consisted of a frequent tone and a rare tone can be interpreted as a statistical learning effect of a sequence with a rare tone.

In the single attention session, the participants were instructed to listen to one of the two simultaneous sequences and ignore the other sequence. In the dual attention session, the participants were instructed to listen to both of the two simultaneous sequences. As a result, in the single attention session, the neural responses to the dyad that consisted of two rare tones in both the attended and ignored sequences were significantly increased compared with those that consisted of two frequent tones in both the attended and ignored sequences. The chord that consisted of two rare tones in both the attended and ignored sequences evoked the highest amplitude of the four types of chords. The chord that consisted of a rare tone in the attended sequence and a frequent tone in the ignored sequence evoked the second highest amplitude of the four. The chord that consisted of a rare tone in the ignored sequence and a frequent tone in the attended sequence evoked the third highest amplitude of the four. The chord that consisted of two frequent tones in both the attended and ignored sequences evoked the lowest amplitude of the four. The peak response latency for the chords that consisted of a rare tone in the ignored sequence and a frequent tone in the attended sequence were significantly shorter compared with those for the dyads that consisted of a frequent tone in the ignored sequence and a rare tone in the attended sequence. These results suggest that statistical learning was facilitated in attentional learning but not in nonattentional learning, which is in agreement with the results of our previous study^13^.

In contrast, no significant statistical learning effect was detected in the dual attention session. The findings in the single and dual attention sessions were also consistent with the behavioural data. Our results suggest that the statistical learning of two simultaneous auditory sequences may be facilitated by paying attention only to one sequence (i.e., attentional learning) and ignoring the other sequence (i.e., nonattentional learning), whereas the learning effect could not be detected when paying attention to both sequences. This hypothesis might be consistent with previous studies^19^. Attentional access to much of the information that occurs concurrently could interfere with the acquisition of this information because cognitive capacity is limited in humans. In natural auditory environments, however, learners can concurrently acquire a great deal of information through both attentional and nonattentional processes. Earlier studies suggested that the brain regions and activation patterns engaged during attentional and nonattentional learning might be partially distinct^21–25^. It has been reported that the neural basis for the spatial dichotomy that underlies attentional and nonattentional learning predominantly depends on the frontal lobe and the striatum, respectively^26–29^. In other words, there might be a specific cognitive capacity underlying nonattentional learning that is independent of the capacity underlying attentional learning. A previous study behaviourally demonstrated that attentional and nonattentional learning operates independently and in parallel when learners were presented with two simultaneous streams of stimuli^19^. Our neurophysiological findings are consistent with those of the previous study. The attentional and nonattentional statistical learning of two simultaneous auditory sequences was reflected in P1 responses, which have been considered to be generated in the vicinity of the primary auditory cortex. When humans have learned transitional probabilities in an auditory sequence, they can predict a forthcoming tone that will frequently follow preceding tones in the sequence. Prediction of forthcoming tones that will appear with higher transitional probability reduces P1 responses in the auditory cortex. Through biased attention in the present study, participants might be able to clearly distinguish and better predict each sequence. We could not, however, demonstrate a difference in the neural basis underlying nonattentional and attentional statistical learning because of the methodological limitations of this study. Further research is needed to clarify the neural substrates for nonattentional and attentional learning.

Previous studies suggest that statistical learning can be reflected in the late components such as N1 and P2 and also in the earlier component, P1^10–15, 17^. Previous studies reported that learning effects on P1 were not correlated with the other event-related responses^30–32^. Some studies suggest that the learning effect relationship with P1 involves music expertise and specialised training experience^33, 34^. Paraskevopoulos *et al*. demonstrated that, in the initial phase of statistical learning, learning effects on P1, but not N1, were larger in musicians compared with non-musicians^12^. In our previous study, the statistical learning of chord sequences was reflected in P1^16^. Another study reported that, in learning the chord progression with conditional probability, the learning effects on the later responses such as early anterior negativity (EAN: 150–250 ms)^35, 36^ were facilitated by musical training^37^. Especially in the initial learning phase such as statistical learning, earlier responses of the P1 may be more associated with perception of musical sequences compared with other components.

In the neurophysiological studies using the paired-click paradigm, the P1 responses are measured as a marker of sensory gaiting function^38^. Compared to single sine tones, the click tones contain a broad frequency spectrum and recruit more neural activity. In the present study, the complex tones may have helped elicit the P1 responses, otherwise showing relatively high inter-individual variability among other components. In addition, infinite averaging of continuous data at every SOA of 0.5 s eliminates signals below a frequency of 2 Hz. The relatively short SOA of 0.5 s corresponding to the applied high-pass filter of 2 Hz may be critical to refractory recovery of the late components such as P2.

Neuroimaging studies have shown that the P1 and N1 components are generated in the auditory cortex with different topographies^39, 40^. P1 and N1 are generated in the lateral part of the primary auditory cortex and the secondary auditory cortex, respectively. Neurophysiological effects of statistical learning on neural responses in the auditory cortex can also be explained in the framework of predictive coding in a top-down manner^18^. The brain constantly generates probabilistic predictions of what is going to happen. The auditory input is compared with the expected tone and produces a signal that codes a prediction error. The lower the probability of the sensory input, the greater the prediction error and reaction to the stimulus. When no error occurs, there is a suppression of responses encoding prediction error in the primary auditory cortex. According to previous studies, earlier auditory responses that peaked at 20–80 ms, which is around P1 latency, were attributed to parallel thalamo-cortical connections, or to cortico-cortical connections between the primary auditory cortex and the superior temporal gyrus^38^. Thus, an early component of auditory responses in lower cortical areas can be interpreted as the transient expression of prediction error that is suppressed by predictions from higher cortical areas in a top-down connection^18^. This suppression may be compromised if the sequences have not been learned previously. The difference in the behaviour of the P1 and N1 responses in statistical learning suggests that the neural basis of the P1 and N1 components reflecting auditory statistical learning is at least partially different. Further studies are needed to clarify the specific attributes of P1 in statistical learning.

In conclusion, we demonstrated that the statistical learning of two simultaneous auditory sequences might be facilitated by paying attention to only one sequence and ignoring the other sequence, whereas the learning effect could not be detected when paying attention to both sequences. Our results suggest that there could be a partially distinct neural basis underlying nonattentional and attentional statistical learning. Biased attention may be an essential strategy under conditions where learners are exposed to multiple information streams.

## Experimental procedure

### Participants

Fifteen right-handed (Edinburgh handedness questionnaires; laterality quotient ranged from 57.9–100)^41^ healthy participants with no history of neurological or audiological disorders were included (9 males, 6 females; age range, 24–36 years). No participants had experience living abroad, and no participants possessed absolute pitch. This study was approved by the Ethics Committee of The University of Tokyo and performed in accordance with the guidelines and regulations. All participants were well informed of the purpose, safety, and protection of personal data in this experiment, and they provided written informed consent for this study.

### Stimuli

#### Tones

Using a cascade-Klatt-type formant synthesizer^42^ HLsyn (Sensimetrics Corporation, Malden, MA, USA), we generated eight complex tones that consisted of fundamental frequencies (F0) in a five-tone equal temperament (F0 = 100 × 2^(n−1)/5^ Hz). The nine tones consisted of 4 low and high pitches each (low pitch: n = 1–4; 100, 115, 132, and 152 Hz, high pitch: n = 11–14; 400, 459, 528, and 606 Hz). Only F0 s were variable; all of the other parameters were constant (duration, 350 ms; rise/fall, 10/150 ms; binaural presentation, 80 dBSPL intensity).

#### Sequences

The auditory stimulus sequence was 728 repetitions of two-tone chords (dyads), each of which consisted of a low and high pitch. Within each chord, the intervals were separated by more than an octave, and they were presented with a stimulus onset asynchrony (SOA) of 500 ms. The order of low and high voice in the dyads was defined according to second-order Markov processes with the constraint that the probability of a forthcoming tone was statistically defined (80% for a tone; 6.67% for the other three tones) by the last two successive tones (Fig. 2). The distinct two of four Markov chains shown in Fig. 2 were used in each of the low and high voices, and the use of Markov chains was counterbalanced across participants. The dyad sequences can also be interpreted as two simultaneous sequences that consisted of low- and high-voice sequences (Fig. 1).

### Experimental protocol

Participants completed two sessions: single and dual attention sessions. In each session, exposure to the sequence during MEG measurement was preceded by a behavioural test. The order of the two sessions was counterbalanced across participants to ensure that specific transitional patterns did not interfere with learning in adjacent experimental sessions.

#### Single attention session

Participants were instructed to listen to a sequence (attended sequence) and ignore the other sequence (ignored sequence). The use of attended and ignored sequences in two simultaneous sequences was counterbalanced across participants. To distinguish between ignored and attended conditions, there was a 500 ms silent period that was pseudo-randomly inserted (i.e., SOA 1000 ms) within every set of 40 successive tones in the attended sequence only. Before the session, participants were instructed to raise their right hand at every silent period in the attended sequence and ignore the other sequence. Using these approaches, we confirmed that all of the participants correctly raised their right hand at every silent period in the attended sequences, and that they were continuing to pay attention only to that sequence.

After measuring MEG, participants completed an interview in which they were presented with 30 series of 8 single tones. Participants then reported whether each 8-tone series sounded familiar. The 30 series of 8 tones were categorised into three types, and the presentation order was randomised. In the 10 series, tones were ordered based on the same constraint as the ignored sequence (tone series I). In an additional 10 series, tones were ordered based on the same constraint as the attended sequence (tone series A). In the remaining 10 series, tones were pseudo-randomly ordered (random tone series).

#### Dual attention session

Participants were instructed to listen to both of the two simultaneous sequences. To distinguish between two attended conditions, a 500-ms silent period was pseudo-randomly inserted (i.e., SOA 1000 ms) within every set of 40 successive tones independently in each sequence. Before the session, participants were instructed to raise their right and left hands at every silent period in a sequence and at the other sequences of the two simultaneous sequences, respectively. Thus, we could confirm that all participants correctly raised their hands at every silent period in both sequences, and that they continued to pay attention to each sequence independently.

After the MEG measurement, participants completed an interview in which they were presented with 30 series of 8 single tones. Participants then reported whether each 8-tone series sounded familiar. The 30 series of 8 tones could be categorised into three types, and the presentation order was randomised. In the 10 series, tones were ordered based on the same constraint as the high-voice sequence (high-voice series). In an additional 10 series, tones were ordered based on the same constraint as lower-pitch sequences (low-voice series). In the remaining 10 series, tones were pseudo-randomly ordered (random tone series).

### Measurement

Measurement and analysis were performed as in our previous studies^13, 14, 16, 17^. We recorded MEG signals from participants while they listened to the two simultaneous sequences. Auditory stimuli were sequenced using the STIM2 system (Compumedics Neuroscan, El Paso, TX, USA), and were binaurally delivered to participant’s ears at 80 dBSPL through ER-3 A earphones (Etymotic Research, Elk Grove Village, IL, USA). MEG signals were recorded in a magnetically shielded room, using a 306-channel neuromagnetometer system (Elekta Neuromag Oy, Helsinki, Finland) with 204 planar first-order gradiometers and 102 magnetometers at 102 measuring sites on a helmet-shaped surface that covers the entire scalp. Auditory stimulus-triggered epochs were filtered online with a 0.1 to 200 Hz band-pass filter and were then recorded at a sampling rate of 600 Hz.

### Data analysis

Epochs with artefacts that exceeded 3 pT/cm or 3 pT for any MEG channel were excluded from analyses. Contamination from environmental noise was reduced using the temporally extended signal space separation method with a buffer length of 10 s and a correlation limit of 0.980^43^. To extract learning effects from the neuromagnetic response series, neuromagnetic responses were selectively averaged from the beginning of the latter half of the sequence until the average number was reached twice for each dyad, and was distinguished according to transitional probabilities. In addition to selective averaging, all responses to dyads in the two sequences were averaged for each participant to evaluate the reliability of the evoked response individual components. The averaged responses were filtered offline with a 2 to 40 Hz band-pass. The baseline for magnetic signals in each MEG channel was defined by the mean amplitude in the pre-stimulus period from −100 to 0 ms. The analysis window was defined as 0 to 500 ms.

**Figure 5:**
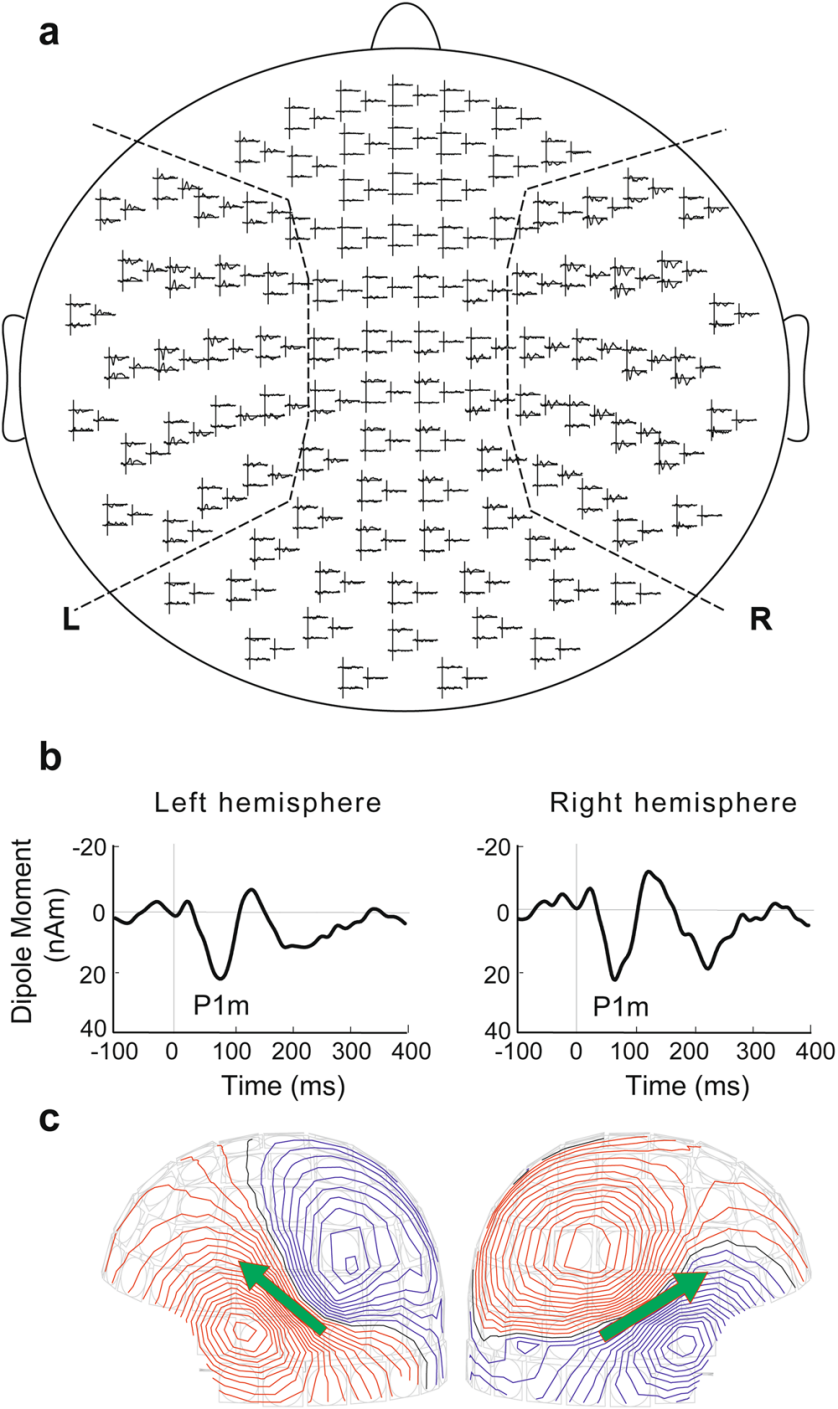
(**a**) The 66 channels selected in each hemisphere for ECD and source-strength calculation are identified using dashed lines. (**b**) Source-strength waveforms for the P1 m responses in a representative subject. The source-strength waveforms for P1 m in each hemisphere were calculated using the ECDs as templates. (**c**) Isofield contour maps for P1 m are shown for each hemisphere. Outflux (red lines) and influx (blue lines) are stepped by 20 fT. The green arrows represent ECDs. The P1 m responses were separately modelled as single attention ECDs in each hemisphere.

The P1 m, N1 m, and P2 m responses were separately modelled as single attention ECDs in each hemisphere. The ECDs for the P1 m, N1 m, and P2 m responses to all dyads were separately estimated at the peak latency using 66 temporal channels for each participant (Fig. 5a). Participants who demonstrated poor ECD estimation, with a goodness-of-fit below 80% in either the left or right hemisphere, were not used in further analysis. The number of participants who demonstrated ECD estimation with a goodness-of-fit above 80% for the P1 m, N1 m, and P2 m components was 12, 5, and 9, respectively. Because there were insufficient samples of N1 m and P2 m for statistical analysis, these components were excluded from further analysis.

The source-strength waveforms for P1 m in each hemisphere were calculated using the ECDs as templates (Fig. 5). Then, in the single attention session, we performed a 2 (hemisphere: right and left) × 4 (stimulus: chord consisting of two frequent tones both in the attended and ignored sequences; chord consisting of two rare tones in both the attended and ignored sequences; chord consisting of a frequent tone in the attended sequence and a rare tone in the ignored sequence; and chord consisting of a rare tone in the attended sequence and a frequent tone in the ignored sequence) repeated-measures ANOVA with peak amplitude and latency of source-strength of P1 m in the time window of 30 ms–90 ms. When we detected significant effects, Bonferroni-corrected post-hoc tests were conducted for further analysis. In the dual attention session, we performed a 2 (hemisphere: right and left) × 4 (stimulus: chord consisting of two frequent tones in both high- and low-voice sequences; chord consisting of two rare tones in both the high- and low-voice sequences; chord consisting of a rare tone in the high-voice sequence and a frequent tone in the low-voice sequence; and chord consisting of a rare tone in the low-voice sequence and a frequent tone in the high-voice sequence) repeated-measures ANOVA with peak amplitude and latency of source-strength of P1 m. When we detected significant effects, a Bonferroni-corrected post-hoc test was conducted for further analysis. In the behavioural test, logit transformation was applied to normalize the familiarity ratios (ratios of answering that the tone series sounded familiar to the number of tone series). We performed an ANOVA using the logit values of the familiarity ratios for each series. When we detected significant effects, Bonferroni-corrected post-hoc tests were conducted for further analysis. Statistical significance levels were set at *p* = 0.05. We could not include between-session factors (i.e., single and dual attention sessions) in the statistical analysis of MEG and behavioural responses, because simultaneous sequences were divided into attended and ignored sequences in the single attention session, and high- and low-voiced sequences in the dual attention session.

## References

[1] Jimenez L, Castor M (1999). Which Attention Is Needed for Implicit Sequence Learning?. Journal of Experimental Psychology: Learning, Memory, and Cognition..

[2] Aizenstein HJ (2004). Regional brain activation during concurrent implicit and explicit sequence learning. Cerebral Cortex..

[3] Saffran JR, Aslin RN, Newport EL (1996). Statistical learning by 8-month-old infants. Science..

[4] Perruchet P, Pacton S (2006). Implicit learning and statistical learning: one phenomenon, two approaches. Trends Cogn Sci..

[5] Frost RL, Monaghan P (2016). Simultaneous segmentation and generalisation of nonadjacent dependencies from continuous speech. Cognition..

[6] Ettlinger M, Margulis EH, Wong PC (2011). Implicit memory in music and language. Frontiers in Psychology.

[7] Francois C, Schön D (2011). Musical expertise boosts implicit learning of both musical and linguistic structures. Cerebral Cortex.

[8] Cleeremans A, Destrebecqz A, Boyer M (1998). Implicit learning: news from the front. Trends in Cognitive Sciences.

[9] Tillmann B, Bharucha JJ, Bigand E (2000). Implicit learning of tonality: a self-organizing approach. Psychological Review.

[10] Abla D, Katahira K, Okanoya K (2008). On-line Assessment of Statistical Learning by Event-related Potentials. Journal of Cognitive Neuroscience.

[11] Furl N (2011). Neural prediction of higher-order auditory sequence statistics. Neuroimage.

[12] Paraskevopoulos E, Kuchenbuch A, Herholz SC, Pantev C (2012). Statistical learning effects in musicians and non-musicians: an MEG study. Neuropsychologia.

[13] Daikoku T, Yatomi Y, Yumoto M (2014). Implicit and explicit statistical learning of tone sequences across spectral shifts. Neuropsychologia..

[14] Daikoku T, Yatomi Y, Yumoto M (2015). Statistical learning of music- and language-like sequences and tolerance for spectral shifts. Neurobiology of Learning and Memory.

[15] Koelsch S, Busch T, Jentschke S, Rohrmeier M (2016). Under the hood of statistical learning: A statistical MMN reflects the magnitude of transitional probabilities in auditory sequences. Scientific Reports.

[16] Daikoku T, Yatomi Y, Yumoto M (2016). Pitch-class distribution modulates the statistical learning of atonal chord sequences. Brain and Cognition.

[17] Daikoku T, Yatomi Y, Yumoto M (2017). Statistical learning of an auditory sequence and reorganization of acquired knowledge: A time course of word segmentation and ordering. Neuropsychologia.

[18] Friston K (2005). A theory of cortical responses. Phil. Trans. R. Soc..

[19] Watanabe K, Funahashi S (2014). Neural mechanisms of dual-task interference and cognitive capacity limitation in the prefrontal cortex. Nature Neuroscience.

[20] Curran T, Keele SW (1993). Attentional and non-attentional forms of sequence learning. Journal of Experimental Psychology: Learning, Memory and Cognition.

[21] Rauch SL (1995). A PET investigation of implicit and explicit sequence learning. Human Brain Mapping.

[22] Poldrack RA, Clark J, PareÂ-Blagoev EJ, Shohamy D (2001). Interactivememory systems in the human brain. Nature.

[23] Reber PJ, Squire LR (1998). Encapsulation of implicit and explicit memory in sequence learning. Journal of Cognitive Neuroscience.

[24] Paradis, M. A Neurolinguistic Theory of Bilingualism. *Amsterdam**:**John Benjamins* (2004).

[25] Destrebecqz A (2005). The neural correlates of implicit and explicit sequence learning: Interacting networks revealed by the process dissociation procedure. Learning & Memory.

[26] Miller EK, Cohen JD (2001). An integrative theory of prefrontal cortex function. Annual Review of Neuroscience..

[27] Corbetta M, Shulman GL (2002). Control of goal-directed and stimulus-driven attention in the brain. Nature Reviews Neuroscience.

[28] Stevens DJ, Arciuli J, Anderson DI (2014). Concurrent Movement Impairs Incidental But Not Intentional Statistical Learning. Cognitive Science..

[29] Daikoku T, Takahashi Y, Futagami H, Tarumoto N, Yasuda H (2017). Physical fitness modulates incidental but not intentional statistical learning of simultaneous auditory sequences during concurrent physical exercise. Neurol Res..

[30] Boutros NN, Belger A (1999). Midlatency evoked potentials attenuation and augmentation reflect different aspects of sensory gating. Biological Psychiatry.

[31] Boutros NN (1995). The P50 evoked potential component and mismatch detection in normal volunteers: Implications for the study of sensory gating. Psychiatry Research.

[32] Kisley MA, Noecker TL, Guinther PM (2004). Comparison of sensory gating to mismatch negativity and self-reported perceptual phenomena in healthy adults. Psychophysiology.

[33] Kizkin S, Karlidag R, Ozcan C, Ozisik HI (2006). Reduced P50 auditory sensory gating response in professional musicians. Brain and Cognition.

[34] Wang W, Staffaroni L, Reid E, Steinschneider M, Sussman E (2009). Effects of musical training on sound pattern processing in high-school students. International Journal of Pediatric Otorhinolaryngology.

[35] Koelsch S, Gunter T, Friederici AD, Schroger E (2000). Brain indices of music processing: “Non-musicians” are musical. Journal of Cognitive Neuroscience.

[36] Koelsch S (2009). Music-syntactic processing and auditory memory: Similarities and differences between ERAN and MMN. Psychophysiology.

[37] Kim, S. G., Kim, J. S. & Chung, C. K. The effect of conditional probability of chord progression on brain response: An MEG study. *PLoS One* **6**, doi:10.1371/journal.pone.0017337 (2011). 10.1371/journal.pone.0017337 PMC3045443 21364895

[38] Adler LE (1982). Neurophysiological evidence for a defect in neuronal mechanisms involved in sensory gating in schizophrenia. Biological Psychiatry.

[39] Liégeois-Chauvel C, Musolino A, Badier JM, Marquis P, Chauvel P (1994). Evoked potentials recorded from the auditory cortex in man: Evaluation and topography of the middle latency components. Electroencephalography and Clinical Neurophysiology.

[40] Yvert B, Crouzeix A, Bertrand O, Seither-Preisler A, Pantev C (2001). Multiple supratemporal sources of magnetic and electric auditory evoked middle latency components in humans. Cerebral Cortex.

[41] Oldfield RC (1971). The assessment and analysis of handedness: the Edinburgh inventory. Neuropsychologia.

[42] Klatt DH (1980). Software for a Cascade/Parallel Formant Synthesizer. Journal of the Acoustical Society of America.

[43] Taulu S, Hari R (2009). Removal of magnetoencephalographic artifacts with temporal signal-space separation: demonstration with single-trial auditory-evoked responses. Human Brain Mapping.

